# Endoscopic Prelacrimal Approach for the Surgical Management of a Late Silicone Foreign Body and an Odontogenic Maxillary Cyst: A Case Report

**DOI:** 10.7759/cureus.103750

**Published:** 2026-02-16

**Authors:** Badr Soudi, Amine Benfaida, Florian Rivieccio, Pierre Louis Chiche, Hassan El Edghiri

**Affiliations:** 1 Otolaryngology - Head and Neck Surgery, Cheikh Zayd University Hospital, Rabat, MAR; 2 Radiology, Mohammed VI University of Health Sciences/Cheikh Khalifa International University Hospital, Casablanca, MAR; 3 Otolaryngology - Head and Neck Surgery, Hôpital de Cannes, Cannes, FRA; 4 Maxillofacial Surgery, Hôpital de Cannes, Cannes, FRA; 5 Otolaryngology - Head and Neck Surgery, Cheikh Zayd Hospital, Rabat, MAR

**Keywords:** caldwell-luc alternative, chronic maxillary sinusitis, endoscopic prelacrimal recess approach, odontogenic maxillary cyst, silicone foreign body

## Abstract

Foreign bodies and odontogenic lesions of the maxillary sinus can lead to chronic rhinosinusitis, particularly when located in compartments that are difficult to access surgically. We report two cases managed using the endoscopic prelacrimal recess approach (EPLA). The first case involved a 76-year-old woman presenting with chronic facial pain and purulent discharge caused by a retained silicone fragment following bilateral inferior turbinectomy performed 20 years earlier. The second case concerned a 28-year-old woman with recurrent left maxillary sinusitis secondary to a 2.1-cm pericoronal cyst associated with an ectopic tooth. In both patients, EPLA allowed direct access to the anterior and inferior maxillary sinus, enabling complete lesion removal while preserving the nasolacrimal duct, natural ostium, and inferior turbinate. Postoperative outcomes were favorable, with complete symptom resolution and no reported complications. These cases demonstrate the feasibility and safety of the endoscopic prelacrimal recess approach for selected maxillary sinus pathologies located in anatomically challenging compartments.

## Introduction

Foreign material or odontogenic lesions within the maxillary sinus can lead to persistent rhinosinusitis and secondary infection, particularly when located in anatomically difficult-to-access compartments. Several surgical approaches have historically been used to address such lesions. The Caldwell-Luc procedure, although once the standard, is associated with significant postoperative morbidity, including facial numbness, oroantral fistula, and chronic pain [1,2]. The middle meatal antrostomy (MMA), as part of functional endoscopic sinus surgery, reduces morbidity but provides limited access to the anterior and inferior regions of the maxillary sinus [3]. The modified Denker approach offers broader exposure of the anterior maxillary sinus but may be associated with increased tissue disruption and altered nasal physiology.

In 2010, Nakamaru et al. introduced the endoscopic prelacrimal recess approach (EPLA) to overcome these limitations [4]. EPLA provides direct access to the anteroinferior maxillary sinus while preserving key structures, including the inferior turbinate and nasolacrimal duct. This approach has since been applied successfully for benign tumors, cysts, and foreign bodies [5,6]. Compared with other techniques, EPLA allows for targeted exposure with minimal disruption of nasal anatomy, though larger studies are required to fully establish its comparative efficacy.

In this report, we assessed the feasibility and outcomes of EPLA in two distinct cases: a chronic silicone foreign body and an odontogenic cyst with an ectopic tooth, lesions that might otherwise require more invasive external approaches.

## Case presentation

Case 1: Silicone foreign body

A 76-year-old woman presented with chronic right maxillary sinusitis and facial pain. Her surgical history included a bilateral partial inferior turbinectomy performed 20 years earlier. She also had diabetes, hypercholesterolemia, and coronary artery disease treated with a stent.

**Figure 1:**
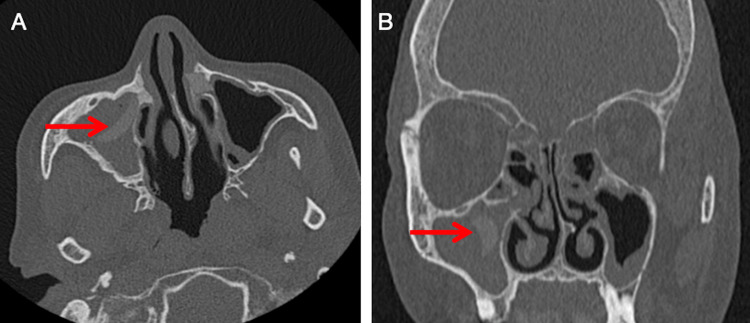
Referenced in Case 1, Diagnostic Assessment section - axial (A) and coronal computed tomography (B) scans showing a hyperdense foreign body (red arrow) in the right maxillary sinus.

Endoscopic examination revealed purulent discharge and bilateral synechiae, more pronounced on the right side. Computed tomography demonstrated complete right maxillary sinus opacification with a well-defined hyperdense foreign body along the lateral wall, consistent with a non-metallic material measuring approximately 15 mm (Figure 1, axial and coronal views).

**Figure 2:**
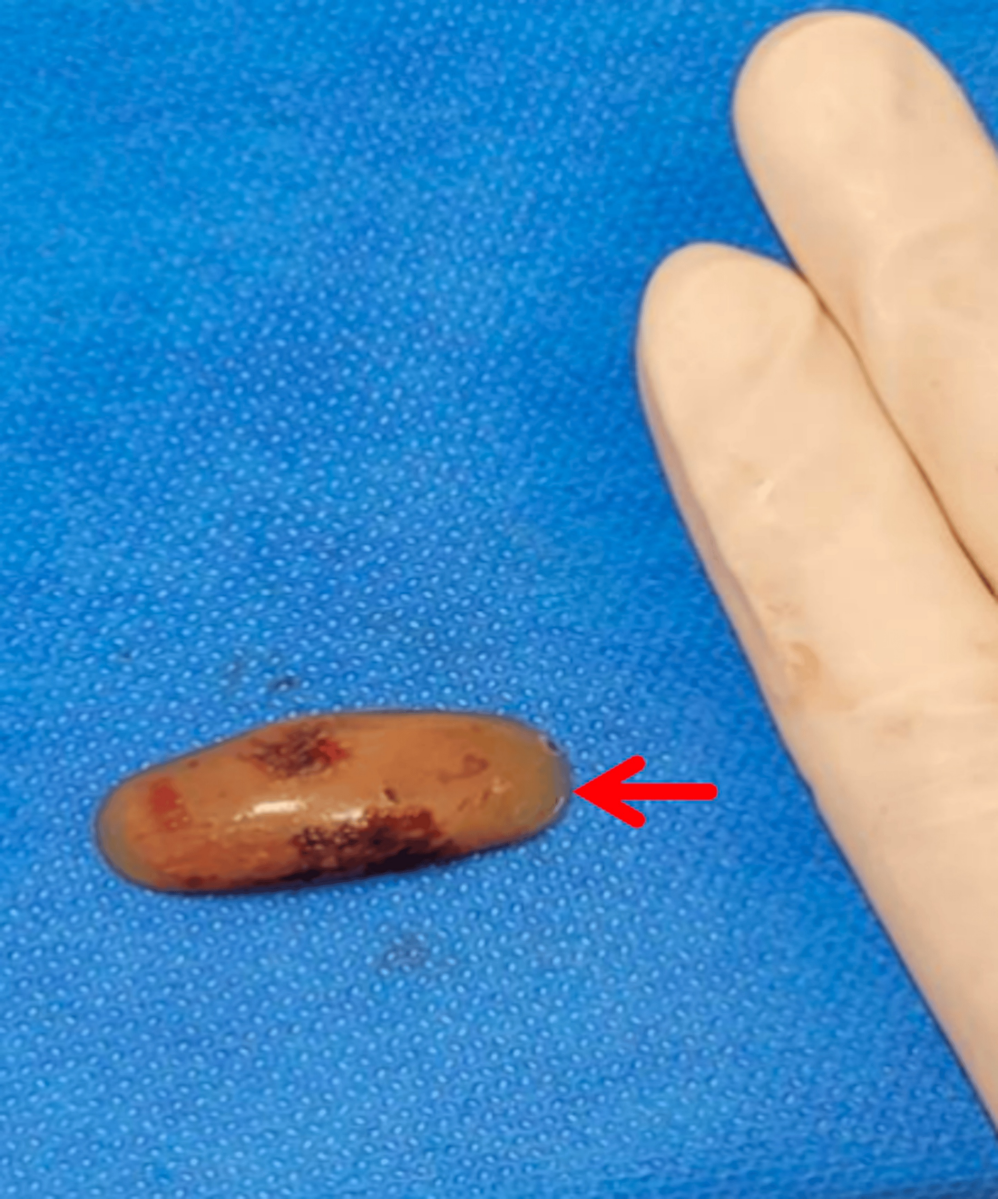
Referenced in Case 1, Therapeutic Intervention section - silicone fragment removed en bloc during surgery (red arrow).

Under general anesthesia, we performed the EPLA. A vertical incision was made anterior to the uncinate process, and the inferior turbinate-nasolacrimal duct (IT-NLD) flap was elevated to expose the anteromedial maxillary sinus. The silicone fragment was dissected and removed en bloc (Figure 2). The flap was repositioned and sutured with absorbable material. No intraoperative images are available for this case.

Histopathologic examination revealed chronically inflamed mucosa without granulomatous reaction. The patient was discharged on postoperative Day 1. At six weeks and three months, she reported complete pain relief and no recurrent discharge.

Case 2: Odontogenic cyst with ectopic tooth

**Figure 3:**
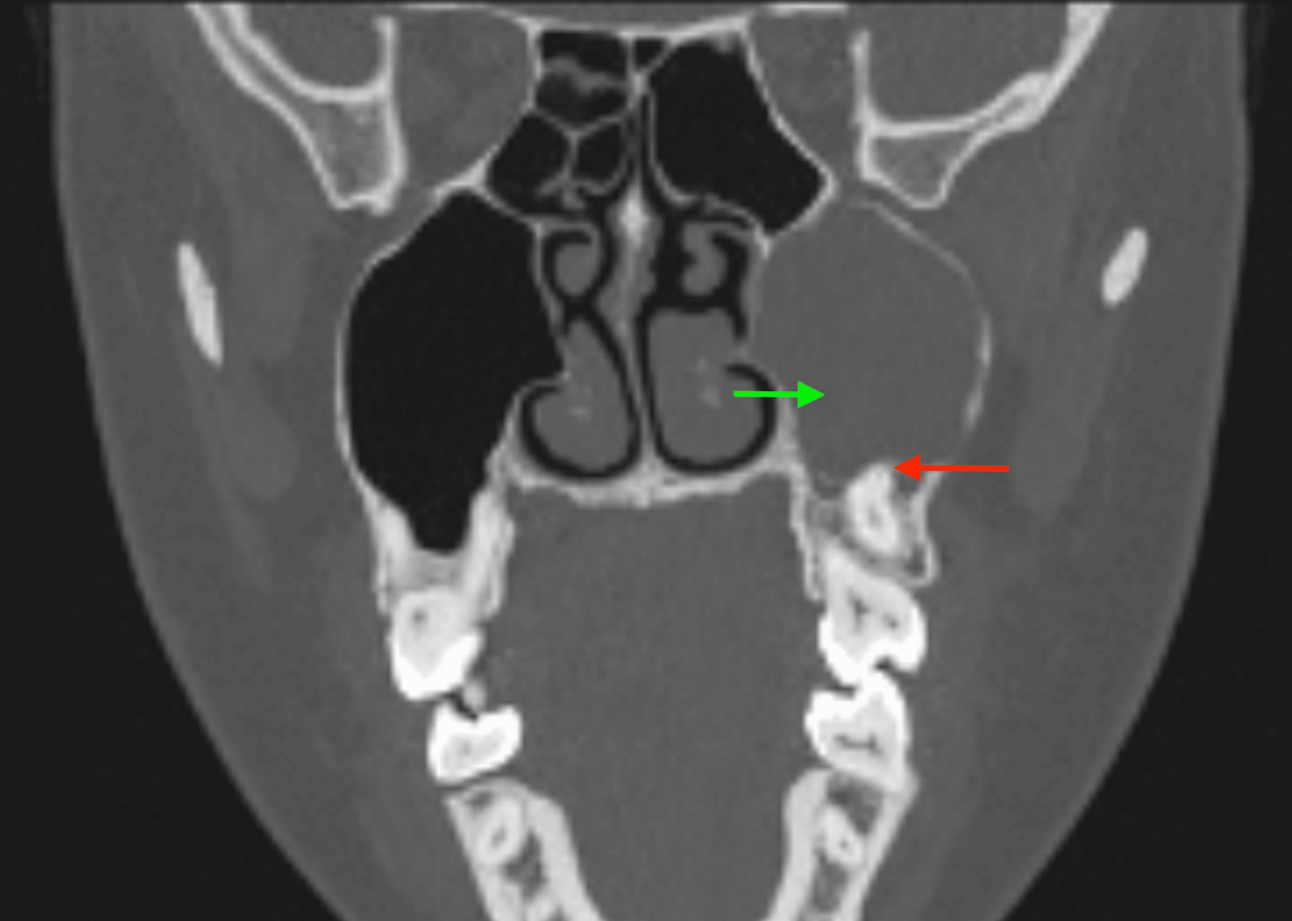
Coronal computed tomography scan showing the pericoronal cyst (green arrow) with an ectopic tooth (red arrow).

A 28-year-old woman presented with recurrent left maxillary sinusitis and dental pain refractory to antibiotics. Nasoendoscopy revealed mucopurulent drainage from the left middle meatus. CT imaging identified a 2.1-cm cyst containing an ectopic tooth in the left maxillary sinus near the natural ostium (Figure 3, coronal view).

Under general anesthesia, we performed EPLA. After an incision anterior to the uncinate process, the IT-NLD flap was raised to access the prelacrimal recess. The cyst and ectopic tooth were removed en bloc under endoscopic control, and the flap was replaced and sutured. Consultation with an oral surgeon was obtained before the procedure to ensure comprehensive management. No intraoperative images are available for this case.

Histological examination confirmed a cyst lined by non-keratinized squamous epithelium with mild chronic inflammation. The patient was discharged the same day. At six months, she remained symptom-free with complete mucosal healing.

## Discussion

Chronic sinusitis secondary to retained foreign material or odontogenic pathology remains a surgical challenge. In the first case, the retained silicone fragment likely originated from previous partial inferior turbinectomy material and induced chronic inflammation years later. Synthetic material within the sinus can perpetuate bacterial colonization and lead to mucosal fibrosis [7-9]. Awareness of such iatrogenic causes is crucial to prevent long-term complications.

Odontogenic disease accounts for up to 12% of maxillary sinus pathology, whereas ectopic teeth within the sinus remain exceptional [10]. These cystic or dental-related lesions can obstruct normal sinus drainage and sustain recurrent infections. Collaboration between otolaryngologists and oral surgeons is essential to ensure complete removal and reduce the risk of recurrence, as demonstrated in the second case.

Several surgical approaches are available for the management of maxillary sinus pathology. The Caldwell-Luc procedure, historically the standard, provides wide access but is associated with postoperative complications such as facial numbness, oroantral fistula, and prolonged recovery [1,2]. MMA reduces morbidity but offers limited visualization of the anterior and inferior sinus compartments [3]. The modified Denker approach allows extensive exposure of the anterolateral maxillary sinus and floor, yet it is more invasive and may carry a higher risk of facial swelling and dental complications [11].

The EPLA broadens access beyond the limitations of MMA while avoiding the morbidity of more invasive techniques. By entering through the prelacrimal recess and elevating the inferior turbinate-nasolacrimal duct flap, the surgeon achieves direct visualization of the anterior and inferior maxillary sinus. This approach preserves physiologic drainage pathways, reduces postoperative discomfort, and minimizes complications such as facial numbness, oroantral fistula, and chronic pain [2,5]. In both patients, EPLA allowed complete visualization and en bloc excision under controlled endoscopic vision.

It is important to note that this report includes only two cases, which represent a limitation. While the outcomes were favorable, larger case series or comparative studies are necessary to further assess the safety, efficacy, and relative advantages of EPLA compared with other approaches.

Overall, EPLA can be considered a minimally invasive option for selected maxillary sinus lesions located in anatomically challenging compartments, particularly when preservation of key structures is desired.

## Conclusions

We report two uncommon cases of maxillary sinus pathology successfully managed using the EPLA: a silicone foreign body of uncertain origin and a pericoronal cyst associated with an ectopic tooth. Both patients experienced complete symptom resolution. EPLA allows excellent exposure of the anterior and inferior maxillary compartments while preserving the nasolacrimal duct, natural ostium, and inferior turbinate. Reported complications are rare and usually transient.

Close collaboration between ENT and dental specialists is essential to optimize the management of complex odontogenic sinus disease. It is important to note that this report describes only two cases, which represents a limitation. While EPLA was effective in these cases, larger case series or comparative studies are necessary to fully evaluate its safety, efficacy, and potential advantages relative to alternative surgical approaches such as the modified Denker or Caldwell-Luc procedures.

Overall, EPLA can be considered a reliable and minimally invasive option for selected maxillary sinus pathologies, particularly in anatomically challenging compartments.
